# Validation of the Questionnaire on Sexual and Reproductive Health Literacy for Adolescents Age 15 to 19 Years in Lao People's Democratic Republic

**DOI:** 10.3928/24748307-20220207-01

**Published:** 2022-01

**Authors:** Viengnakhone Vongxay, Souksamone Thongmixay, Lianne Stoltenborg, Amphone Inthapanyo, Vanphanom Sychareun, Kongmany Chaleunvong, Dirk Rombout Essink

## Abstract

**Background:**

Beyond sexual and reproductive health (SRH) knowledge, it is sexual and reproductive health literacy (SRHL) that reflects the capacity to deal with sexuality. Many interventions have been conducted to increase SRH knowledge in adolescents, but SRHL has rarely been measured, and a well-validated tool is needed to measure it. Objective: This study aimed to validate a tool to measure adolescent SRHL.

**Methods:**

Reliability, validity, and cultural equivalence were investigated using data from expert consultations, cognitive interviews, and two-pilot studies. Then adaptation was made to the SRHL questionnaire for correct use among Southeast Asian adolescents in Lao and in wider groups.

**Key Results:**

The SRHL tool was comprised of 39 question items focusing on teenage pregnancy, contraception, and abortion. Conceptual, item, and semantic equivalence were all met. Interviewer-administrated mode was found to be optimal. Each question offers the answer choices *very difficult*, *difficult*, *easy*, and *very easy*, with a good to excellent Cronbach's alpha (0.8–0.9); there were no missing items and no floor/ceiling effects. Construct validity was high as 6 of 7 hypotheses were confirmed.

**Conclusion:**

Validation was completed with good cross-cultural validity. The tool was shown to be effective in determining the level of SRHL in adolescents in Laos and potentially in other countries with similar cultures. [***HLRP: Health Literacy Research and Practice*. 2022;6(1):e37–e50.**]

**Plain Language Summary:**

To find out how much adolescents know about sexual and reproductive health, an appropriate instrument of measurement is needed. Using different methods, we investigated the performance of a new tool, namely the SRHL questionnaire, which has 39 questions and should be used with an interviewer to assist in recording responses. This new tool could be used effectively to determine the level of literacy on sexual and reproductive health among adolescents.

Health literacy (HL) is fundamental to improve the well-being of individuals, families, and communities to reduce health inequities (Duong et al., 2017). “Health literacy is linked to literacy and entails people's knowledge, motivation and competence to access, understand, appraise, and apply health information in order to make judgments and take decisions in everyday life concerning healthcare, disease prevention and health promotion to maintain or improve quality of life during the life course” (Sørensen et al., 2012, p. 3). The definition indicates that health-related knowledge is a part of HL, but that HL goes beyond knowledge. In this article, we address sexual and reproductive health literacy (SRHL), which applies the HL concept more specifically to sexual and reproductive health (SRH)—the physical, mental, and social well-being related to the reproductive system and sexuality (UNFPA, 2018a). Higher SRHL levels are associated with safer sexual behaviors (World Health Organization [WHO], 2017a), and adolescents with higher SRHL are less likely to become pregnant unintentionally or to experience maternal morbidity and mortality (Braine, 2009).

SRHL is low in Lao People's Democratic Republic (PDR) (Vongxay et al., 2019). The country has the highest teenage pregnancy (TP) rates in Southeast Asia; 18.4 % of Lao women become mothers before age 18 years (National Statistics Bureau, 2018). TP is associated with infant prematurity, low birth weight, and perinatal and maternal death (Ganchimeg et al., 2013). Each week, two Lao mothers who are younger than age 19 years die as a result of pregnancy-related problems (UNFPA, 2018b), and 23.2% of sexually active young women reported that they had undergone an abortion (UNFPA, 2008). TP can also lead to negative effects on girls' education, employment, and social participation (UNFPA, 2018c).

One factor underlying the high pregnancy rate is the low SRHL among Lao adolescents. In 2017, an SRHL tool was developed to measure the SRHL level among Lao adolescents age 15 to 19 years. The SRHL level among respondents was rated as inadequate, with an average index score of 19.2 of a maximum of 50. To develop the SRHL questionnaire, the 47-item version of the European Health Literacy Survey Questionnaire (HLS-EU-Q47) was adapted and made specific to sexual and reproductive health topics (Pelikan et al., 2012). The SRHL questionnaire consists of 45 questions. Adolescents were asked to answer them using a 4-point Likert scale (1 = *very difficult*, 4 = *very easy*) to report on how easy it was to find, understand, judge, or use SRH information. However, at that time the authors also suggested potential improvements to the tool (Vongxay et al., 2019).

Previous studies had suggested that a comprehensive tool should remain valid and reliable with respect to what it intends to measure. It should be developed and approved through a mixed-approach design such as semi-structured in-depth interviews, cognitive interviews, expert consultations, and field testing and re-testing (Dageforde et al., 2015; Herdman et al., 1998; Peters et al., 2014; Tsai et al., 2011). Furthermore, an effective measurement tool should ensure representation—reflect the nature of target users regardless of whether they are single or multiple groups/cultures (Huang & Wong, 2014). Therefore, a validated measurement tool should be general and usable within one or among multiple similar cultures.

The Lao Government has made efforts to improve the HL and to ensure the well-being of adolescents by introducing SRH policies that encourage young people to access family planning and sexual education (SE) and by prohibiting child marriage. However, evaluation of the effect of the policies has been difficult until 2017, since there was no tool to measure whether the young people were aware of them and able to use the services to measure adolescent SRHL. The new SRHL questionnaire offers a great opportunity to measure the progress and to evaluate the effects of future SRH policies. However, the accuracy of the tool still needed to be confirmed in a rigorous process of quality testing, using a mixed-method validation that includes both experts and target users (Dageforde et al., 2015; Herdman et al., 1998; Peters et al., 2014; Tsai et al., 2011) while adapting for cross-cultural validity (Huang & Wong, 2014). Herdman et al. (1998) provide a model to assess cross-cultural equivalence of questionnaires and distinguishes six levels of equivalence: (1) conceptual (relevance of the tool to measure what it intends to measure), (2) item (adequacy of items to measure), (3) semantic (appropriateness of terms used), (4) operational (formulation of item layout and mode of use), (5) measurement equivalence (proof of reliability and psychometric properties), and (6) functional equivalence (well use across cultures).

This article describes the development and validation of a new questionnaire to monitor SRHL among in-school and out-of-school adolescents in Lao PDR, and its correlation to condom literacy skill (as functional literacy on condom information), TP-related knowledge, sexual experience, gender, attitude toward contraception, exposure to SE, and schooling status.

## Methods

### Study Design

An equivalence approach for cross-cultural adaptation of measurement tools was used. This approach was developed by Herdman et al. (1998) and applied by Peters et al. (2014) among others. The model includes six types of equivalence, of which we explicitly use five, and emphasizes that an adaptation process requires input from experts, target users, and field testing. A mixed-method design was chosen to gain insight into the five equivalences: conceptual equivalence assesses whether the tool measures what it intends to measure, how the concept of SRH is conceptualized, which domains are relevant, and the significance accorded to these domains. Item equivalence looks at whether the items are adequately chosen; in other words, whether the items are equally relevant and acceptable in the original and in the new questionnaire. Semantic equivalence looks at the appropriateness of the language used, and how meaning is transferred. Operational equivalence concerns the suitability of the questionnaire format, instructions, and mode of administration. Measurement equivalence looks at proof of reliability and psychometric properties (internal consistency, floor and ceiling effects, and construct validity) of the scale. Herdman et al. (2018) describe a sixth type of equivalence—functional equivalence—which assesses whether the five equivalences together are sufficiently achieved to ensure the questionnaire measures the same thing across cultures. We have not used this type of equivalence. Alternatively, we do describe functional literacy, where we directly assess the skills of participants, to assess whether the questionnaire indeed measures the traits it aims to measure. Herdman et al. (1998) provide a more detailed description of these equivalences.

We were also inspired by Tsai et al. (2011), who used a mixture of qualitative and quantitative methods, especially recruiting HL experts for consultation from the beginning, to generate HL assessment items. The process of the current study design was further inspired by previous tool validation reports (Dageforde et al., 2015; Conrad & Blair, 2009; Flaherty et al., 1988; Huang & Wong, 2014; Nanda et al., 2013; Terwee et al., 2007), which will be referred to in the two phases described below.

This study was conducted in 2018 in Vientiane, the capital of Lao PDR, and consisted of two distinct phases that are described separately below: (1) the questionnaire evaluation phase and (2) the two phases of pilot testing. The original and the adapted questionnaires are added as supplementary files (see https://bit.ly/SRHL2022).

### The Phases of the Study

**Figure 1.**
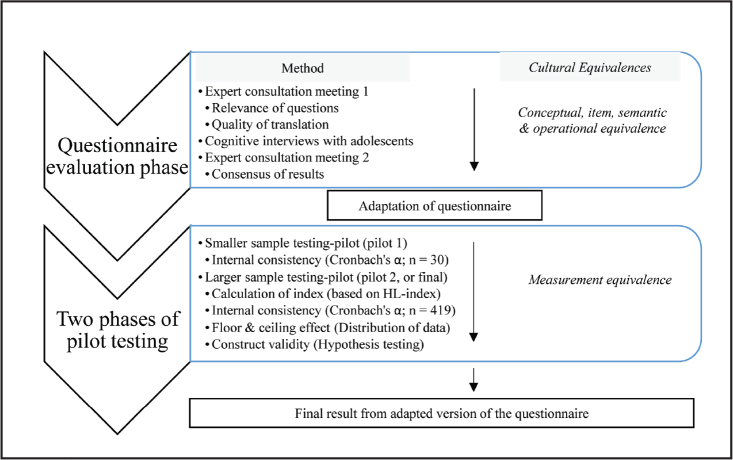
Flowchart of sexual and reproductive health literacy measurement tool validation. HL = health literacy.

Two main phases can be distinguished in the process of adaptation; the questionnaire evaluation phase and the two-pilot testing phase. In the questionnaire evaluation phase, we mainly addressed conceptual, item, semantic, and operational equivalence, by first consulting experts, followed by cognitive interviews with adolescents, and finally another expert meeting to integrate findings and to finalize the questionnaire for piloting. In the two-pilot testing phase, we assessed measurement equivalence by first conducting a small pilot with 30 adolescents and thereafter a larger pilot with 419 participants. We illustrate the process in **Figure 1**. The methods within each phase are described in more detail below.

## Questionnaire Evaluation Phase

### Participants

Experts for consultation meetings were purposively selected based on specific criteria, such as working in the area of, or closely related to, sexual and reproductive health, research methodology, biostatistics, and adolescent programs (**Table 1**). Invitations were sent to seven selected experts 1 week before the meeting; all of them accepted to participate. Adolescents for the cognitive interviews were purposively selected, based on age (15–19 years), representing both in-school and out-of-school groups, and living inside and outside of the city. Ten adolescents, six in-school and four out-of-school, were recruited for the cognitive interviews by purposive selection. Adolescents still attending school were selected from two upper secondary schools, located in an urban and a peri-urban district of Vientiane. Out-of-school adolescents were recruited at a garment factory in a peri-urban district. Contacting the adolescents was facilitated by the local chief of youth groups and using peer-to-peer contact at each site.

**Table 1:**
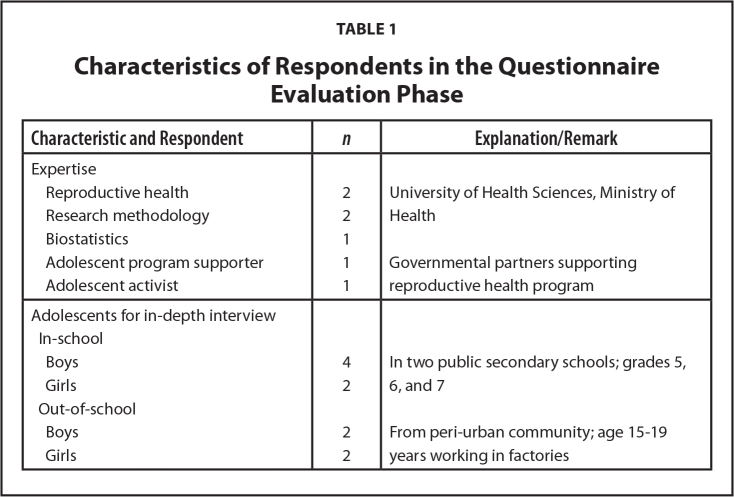
Characteristics of Respondents in the Questionnaire Evaluation Phase

**Characteristic and Respondent**	***n***	**Explanation/Remark**

Expertise		
Reproductive health	2	University of Health Sciences, Ministry of Health
Research methodology	2	
Biostatistics	1	
Adolescent program supporter	1	Governmental partners supporting reproductive health program
Adolescent activist	1	

Adolescents for in-depth interview		
In-school		
Boys	4	In two public secondary schools; grades 5, 6, and 7
Girls	2	
Out-of-school		
Boys	2	From peri-urban community; age 15–19 years working in factories
Girls	2	

### Process and Measures

In the evaluation phase (**Figure 1**), two consultation meetings with the same seven experts and ten semi-structured cognitive interviews with adolescents were organized to evaluate the five equivalences of the questionnaire. The first expert meeting was a semi-structured discussion covering the relevance of the questions, the quality of the translation, the cultural appropriateness, the terms and wording, structure, response alternatives, order of questions, instructions for administration, layout, and any typographical errors (Herdman et al., 1998; Nanda et al., 2013). At the meeting, experts were asked to give an opinion: “What do you think this questionnaire tries to measure? How is it related to SRH in adolescents?” The discussion continued for 120 minutes.

Cognitive interviews with adolescents were conducted after the first expert meeting to check whether it was valid to use with adolescents based on their own perspectives/interactions. Adolescents were asked to think aloud; they were asked to report on the mental processes they used to produce an answer (Conrad & Blair, 2009). The interviewer probed for the respondents' definition of terms or concepts (such as, “What does the term unwanted pregnancy mean to you?”) or interpretation of the question (for example, “Can you tell me in your own words what this question means to you?”). Interviews were expected to provide insight into the conceptual, item, semantic, and operational equivalence. Adolescents were also asked about the structure and administration mode of the questionnaire. The average interview duration was 57 (±5) minutes (range, 50 mn-65 mn). After each interview, question items were adapted, and the new version tested in the next interview. The revised questionnaire was pilot-tested with 30 in-school and out-of-school adolescents to examine the internal consistency of questions. The second expert meeting was then organized to discuss the above-mentioned topics, considering the responses of the adolescents. The following topics were assessed.

***Conceptual equivalence.*** Conceptual equivalence was the means to ensure that the tool was developed to measure what it really intends to measure. Experts were asked to rate the questionnaire items prior to the meeting, to assess the content validity. The content focused on pregnancy, contraception, and abortion in teenagers.

***Item equivalence.*** Item equivalence was to make sure that the items as formulated in the questionnaire could be used, were not too long or too short, and sufficiently captured the content of interest. All items of the SRHL questionnaire were reviewed and rated as favorable (+) or unfavorable (−) for relevance, representation, and comprehensibility. The ratings were discussed during the expert meeting. Items that were rated differently by the experts were discussed until consensus was reached on a rating for each item.

***Semantic equivalence.*** Semantic equivalence was an important step to ensure that the terms employed in the questionnaire were close enough in meaning to the understanding of users, especially the respondents. Three steps were performed: back-and-forth-translation (English-Lao-English) by one independent translator, translation consensus during the expert meeting, and cognitive interview with selected adolescents in and outside of school. For the translation quality, the level of semantic equivalence of the Lao and English version was discussed. The Lao version was back translated to English by a bilingual, independent, native-born Lao lay person, without any prior knowledge of the original questionnaire. Subsequently, the equivalence was scored by experts on a 3-point scale as 1 = *different meaning* in each version, 2 = *almost the same meaning in both versions*, and 3 = *exactly the same meaning in both versions* (Flaherty et al., 1988). Items that were rated as different were excluded or revised. Rewording had priority over excluding because the questionnaires' psychometric properties needed to be considered. Items that were rated as *almost the same* were reconsidered until consensus was reached (Flaherty et al., 1988).

***Operational equivalence.*** Operational equivalence was needed to make sure that usage was suitable to the targeted users. Questionnaire format, order of questions, response alternatives, instructions, and the mode of administration were all discussed in the expert consultation meeting.

### Data Analysis

A framework-based analysis was used to analyze the interviews. First, the records from two expert meetings and ten cognitive interviews were transcribed and translated from Lao to English. Second, the transcripts were summarized per question. Third, the information obtained was structured based on the four themes of the cross-cultural equivalence testing framework: conceptual, item, semantic, and operational equivalence (Nanda et al., 2013). Subthemes of operational equivalence were layout, mode of administration, and answering scale. Last, participants' quotes were compared within each theme/subtheme to determine the cultural equivalence between the original and adapted questionnaire versions. To investigate measurement equivalence, psychometric properties were examined and discussed in the section on two-pilot testing.

## Two Phases of Pilot Testing

Two phases of pilot testing were conducted to examine measurement equivalence (construct validity, internal consistency, and floor and ceiling effects). The statistical reliability of the adapted questionnaire was assessed between a smaller and a larger group of adolescents. This was a new way to look at the reliability of the questionnaire by expecting equal, or nearly equal, statistical reliability between different sizes of sample and in different participants; test-retesting would have been difficult because of obstacles in recruiting the same adolescent participants in the study context, especially the out-of-school adolescents.

By this time, after the expert discussions, the tool was clear that its main focus was on TP and closely related topics. Construct validity was tested by examining a pre-defined hypothesis. The aim was extended to examining the associations of SRHL with condom literacy skill, TP-related knowledge, sexual experience, attitude toward contraception, exposure to SE, and schooling status.

### Participants

For the first pilot testing, 30 in-school and out-of-school adolescents age 15 to 19 years were recruited by multiple sampling techniques. Students in a public secondary school who had never been included in a previous survey were purposively selected. Six to seven adolescents were selected randomly from classes with an average of 30 students by a teacher in charge of youth activities in the school. Student recruitment was done during a free hour or after lectures. Out-of-school adolescents working in a shopping mall and factory; they were recruited by convenience, without a sampling frame, but identifying targets on site and asking if they were age 15 to 19 years and willing to participate. Participant selection included attention to recruiting both boys and girls.

For the second pilot test, 419 in-school and out-of-school adolescents who were age 15 to 19 years were recruited. This age group is normally in grades 5, 6, and 7 of secondary school in Laos. In-school adolescents were recruited by random sampling at public secondary schools in nine districts of Vientiane city, excluding the school where the first pilot testing was done. In each selected school, three classrooms of upper secondary level, grades 5, 6, and 7, were randomly selected and all adolescents in each selected classroom were recruited for the pilot. Out-of-school adolescents were recruited by purposive and snowball sampling from three factories outside the city, in the community, and shopping malls. We selected these sites because of the out-of-school adolescents who were age 15 to 19 years and likely to be available. After recruiting one person at each site, we used snowball sampling, asking one participant to help find others of the same age group. Participants in the first pilot testing were excluded.

### Measures

The tools in this study were the original SRHL questionnaire (self-administered version) of Vongxay et al. (2019), scoring sheets for evaluation of relevance and translation quality of questions, semi-structured guide for cognitive interviews, and adapted SRHL questionnaire (interviewer-administered version). The SRHL items are in the full set questionnaire, which consists of five parts: (1) sociodemographic information, (2) personal health information, (3) information sources, knowledge, behavior, and attitude related to TP, contraceptives, and abortion, (4) SRHL, and (5) functional literacy (see S5: https://bit.ly/SRHL2022); the SRHL items are in Part 4.

For the functional HL (see Part 5), we considered the condom literacy skill of individuals. The set of question items in this part was adopted from a study (Vongxay et al., 2019) that was validated, tested, and first used in 2017 in Laos. We instructed the trained interviewers to give a condom box to each respondent to look at and have them check the information. Then the interviewer asked the listed questions (see S5: https://bit.ly/SRHL2022) but did not ask them to demonstrate the practice of putting a condom on any object shaped like the male sexual organ (because the experts considered it was ethically inappropriate). The explaining about the condom package was a more acceptable way to check related functional literacy, asking the person to obtain information from the condom package, which was assessed based on scoring of right and wrong answers to each question by the investigator based on the response and summing to give the score). This part of the questionnaire was included to investigate the interaction between SRHL and condom literacy (see the hypothesis).

### Analysis of Pilot Testing

Data from both pilot tests were entered and analyzed to examine the internal consistency of questions, using IBM SPSS Statistics 24.0. A Cronbach's alpha of above 0.7 with no missing values and no floor and ceiling effect were expected to indicate the reliability of use for the adapted questionnaire. Floor and ceiling effects are present if ≥15% of the respondents answer the questionnaire with the lowest or highest possible score (Terwee et al., 2007).

In the second pilot test, construct validity was assessed. Seven statistical hypotheses were tested to see how the SRHL index was associated with key characteristics of target users, based on a previous study (Vongxay et al., 2019). These included condom literacy skill, TP-related knowledge, attitude toward contraception use by adolescents, exposure to sex education, schooling, gender, and sex experience. The analysis will also show the strength of the adapted questionnaire with a good construct and adequate psychometric properties, based on the relationship between SRHL index and selected testing properties described by Vongxay et al. (2019). The hypotheses expected the SRHL index to have (we expected to find): (1) a postiive correlation with condom literacy as a skill (SRHL and skill are positively correlated); (2)a positive correlation with TP-related knowledge (SRHL and knowledge are positively correlated); (3) a positive correlation with a liberal attitude toward contraception in adolescents (SRHL and attitude are positively correlated); (4) an association with exposure to SE (SRHL could be supported by specific education); (5) an association with characteristic of attending school (SRHL is better in school group); (6) no difference of SRHL index between genders (consistent with the original version); and (7) no association with sexual experience (consistent with the original version).

The tests of these seven hypotheses were also to see whether the SRHL index score has a similar association with those seven properties as was found for the original version described by Vongxay et al. (2019). With the concept of HL that links knowledge with motivation and skill, and by statistical testing (Pearson's correlation and *t*-test), we expected associations to be detected for knowledge, attitude, skill, exposure to SE, and schooling status. If the results of hypotheses testing were as expected, it would demonstrate one of the strengths of the adapted questionnaire.

## Ethical Considerations

This study was approved by the University of Health Sciences in Vientiane, Lao PDR (UHS Ethical Clearance, N.001/2017). Participants were informed about the study purpose, that their participation was completely voluntary, and that confidentiality would be maintained. Written informed consent was obtained from all adolescent respondents. The experts provided consent in the form of acceptance of the invitation to participate. Data collection with in-school adolescents was based on school principal approval and organized during students' out-of-class time. For out-of-school adolescents, we asked consent directly from each participant. The Ethical Committee agreed that parental consent was not required for the target group (age 15–19 years). The questions are sensitive for respondents, especially in an educational system where sexual activity is taboo. The ethical committee, however, deemed the method used as non-invasive. However, confidentiality must be ensured, as some questions are personally sensitive (e.g., asking about experience of sex, pregnancy, abortion, and drug addiction). Investigators ensured the confidentiality and appropriateness of the interview environment. A one-page information sheet was given to the adolescents, which described the study purpose and the investigator's contact information should any questions arise.

## Results

In total 7 experts for consultation, 10 adolescents for interviews, 30 adolescents for first pilot testing, and 419 for second pilot testing participated in the study (**Tables 1** and **2**).

**Table 2:**
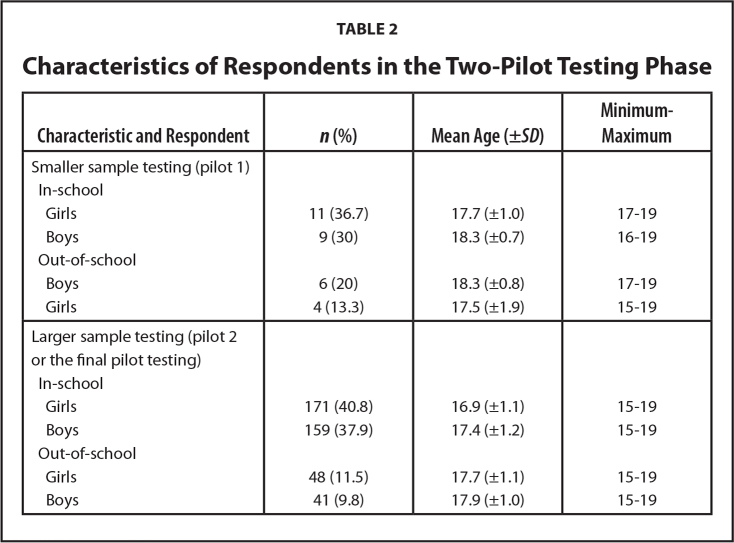
Characteristics of Respondents in the Two-Pilot Testing Phase

**Characteristic and Respondent**	***n* (%)**	**Mean Age (±*SD*)**	**Minimum–Maximum**

Smaller sample testing (pilot 1)			
In-school			
Girls	11 (36.7)	17.7 (±1.0)	17–19
Boys	9 (30)	18.3 (±0.7)	16–19
Out-of-school			
Boys	6 (20)	18.3 (±0.8)	17–19
Girls	4 (13.3)	17.5 (±1.9)	15–19

Larger sample testing (pilot 2 or the final pilot testing)			
In-school			
Girls	171 (40.8)	16.9 (±1.1)	15–19
Boys	159 (37.9)	17.4 (±1.2)	15–19
Out-of-school			
Girls	48 (11.5)	17.7 (±1.1)	15–19
Boys	41 (9.8)	17.9 (±1.0)	15–19

The findings are presented below per equivalence and summarized in **Table 3**.

**Table 3:**
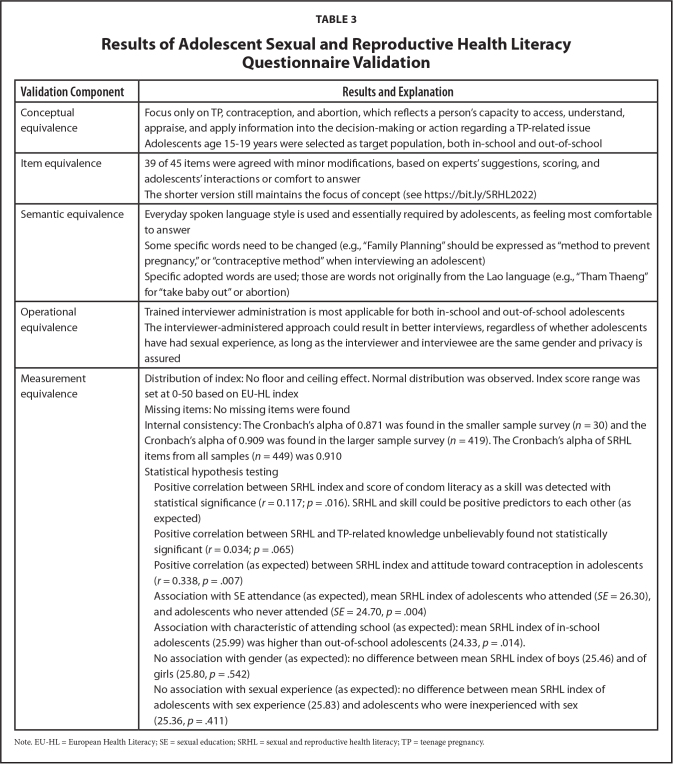
Results of Adolescent Sexual and Reproductive Health Literacy Questionnaire Validation

**Validation Component**	**Results and Explanation**

Conceptual equivalence	Focus only on TP, contraception, and abortion, which reflects a person's capacity to access, understand, appraise, and apply information into the decision-making or action regarding a TP-related issue Adolescents age 15–19 years were selected as target population, both in-school and out-of-school

Item equivalence	39 of 45 items were agreed with minor modifications, based on experts' suggestions, scoring, and adolescents' interactions or comfort to answer The shorter version still maintains the focus of concept (see https://bit.ly/SRHL2022)

Semantic equivalence	Everyday spoken language style is used and essentially required by adolescents, as feeling most comfortable to answer
	Some specific words need to be changed (e.g., “Family Planning” should be expressed as “method to prevent pregnancy,” or “contraceptive method” when interviewing an adolescent)
	Specific adopted words are used; those are words not originally from the Lao language (e.g., “Tham Thaeng” for “take baby out” or abortion)

Operational equivalence	Trained interviewer administration is most applicable for both in-school and out-of-school adolescents The interviewer-administered approach could result in better interviews, regardless of whether adolescents have had sexual experience, as long as the interviewer and interviewee are the same gender and privacy is assured

Measurement equivalence	Distribution of index: No floor and ceiling effect. Normal distribution was observed. Index score range was set at 0–50 based on EU-HL index
	Missing items: No missing items were found
	Internal consistency: The Cronbach's alpha of 0.871 was found in the smaller sample survey (*n* = 30) and the
	Cronbach's alpha of 0.909 was found in the larger sample survey (*n* = 419). The Cronbach's alpha of SRHL items from all samples (*n* = 449) was 0.910
	Statistical hypothesis testing
	Positive correlation between SRHL index and score of condom literacy as a skill was detected with statistical significance (*r* = 0.117; *p* = .016). SRHL and skill could be positive predictors to each other (as expected)
	Positive correlation between SRHL and TP-related knowledge unbelievably found not statistically significant (*r* = 0.034; *p* = .065)
	Positive correlation (as expected) between SRHL index and attitude toward contraception in adolescents (*r* = 0.338, *p* = .007)
	Association with SE attendance (as expected), mean SRHL index of adolescents who attended (*SE* = 26.30), and adolescents who never attended (*SE* = 24.70, *p* = .004)
	Association with characteristic of attending school (as expected): mean SRHL index of in-school adolescents (25.99) was higher than out-of-school adolescents (24.33, *p* = .014).
	No association with gender (as expected): no difference between mean SRHL index of boys (25.46) and of girls (25.80, *p* = .542)
	No association with sexual experience (as expected): no difference between mean SRHL index of adolescents with sex experience (25.83) and adolescents who were inexperienced with sex (25.36, *p* = .411)

Note. EU-HL = European Health Literacy; SE = sexual education; SRHL = sexual and reproductive health literacy; TP = teenage pregnancy.

### Conceptual Equivalence

Experts indicated that the face validity of the questionnaire was inadequate to measure what it intends to measure. SRH is a broad concept, whereas the questionnaire seemed insufficient to measure a status of good or poor SRHL of individuals. The title of the questionnaire, *Sexual and Reproductive Health Literacy,* suggested that it would measure all domains of SRH; therefore, the experts expected the questionnaire to include questions about sexually transmitted diseases, sexual violence, and other topics. We felt that attempting to include all of the topics would make the questionnaire too long and more difficult to grasp. The questionnaire focused on TP, contraceptives, and abortion. Therefore, it was considered more appropriate to reconsider content validity and to have the tool focus on TP and its correlates instead of trying to include all domains of SRH. The experts argued that a focus on TP was relevant and necessary for the context of Lao PDR, because of its high TP rate.

The number of items was then reduced from 45 items to 39. Some items were revised or removed (e.g., “is it difficult or easy to judge which behavior is risky to sexual and reproductive health in your community?”) The SRHL questionnaire was finally considered adequately to measure the aspects related to TP, which indicated sufficient face validity.

Regarding conceptual equivalence, experts indicated that all domains of HL (accessing, understanding, appraising, and applying) are important in the Lao context. In the original version (see S1: https://bit.ly/SRHL2022), the questionnaire structure seemed not to reflect clearly enough the HL concepts closely related to adolescent SRH. Experts recommended to restructure it based on the four HL dimensions. The questions were then reformulated in a more specific and contextual way making it easier to distinguish the different dimensions (see S2: https://bit.ly/SRHL2022).

### Item Equivalence

Adolescents and experts indicated that all 39 items of the adapted SRHL questionnaire were relevant and acceptable in the Lao context, implying item equivalence. These 39 items were the result of revising (excluded/refined) from the 45 items in the original version and achieved consensus after two expert meetings.

### Semantic Equivalence

The quality of the translation was assessed for both the original and the adapted versions during two expert meetings. For both questionnaires, most questions were rated as *almost the same meaning* or *exactly the same meaning*. In the original questionnaire, the translation of the concepts “abortion” and “judging” differed between the Lao and the English versions. One expert mentioned that the meaning of abortion (Lulouk) was unclear in the Lao language. Adolescents thought of abortion as a miscarriage, a termination of the pregnancy due to an accident, or did not have an idea what was meant. Most adolescents preferred the Thai word “Tham thaeng,” which is used for induced abortion in Lao. One respondent said:

“I believe that a safe abortion is when women go to the hospital to get induced [Thamtheng] and unsafe abortion is to take Yachine to get induced.” (Participant 8). Yachine, or translated as Chinese pill, is well known in Laos as a pill for induced abortion.

However, adolescents found it difficult to fully understand these domains and to judge whether they should be included in the questionnaire. Experts and adolescents indicated that this was due to non-transparent translations and too-broad questions. Direct translation of some words in the Appraising part of the older version of the questionnaire (e.g., judging, evaluating) were found to be difficult to use with adolescents. Some questions were phrased in a broad manner (e.g., “. . .Judge how to avoid risky behaviors that lead to unsafe sex?”). Some questions therefore were interpreted and translated into Lao language as “deciding or choosing” (e.g., Decide how to avoid risk behaviors that lead to unsafe sex?) and included in the “applying” part of the questionnaire. Adolescents then felt more confident to answer about judging the quality of information sources (e.g., doctors versus other sources). “Doctors are better, because they are experts. But youth counsellors are just counsellors or volunteers.” (Participant 6)

Based on cognitive interviews, small adaptations were made to the questionnaire to fit with the everyday language of adolescents. Adolescents preferred to use “having a baby” instead of “pregnancy,” “chance to get problems” instead of “risk factors,” and “methods to prevent having a baby” instead of “family planning and/or contraceptives.” Some adolescents understood the word “service” as entertainment places, such as bars or beer shops or where there are girls to provide entertainment or sex. Even referring to the youth-friendly service, instead of calling it that, adolescents preferred to refer to it more generally as health providers in the adapted questionnaire. An adolescent said: “I think that the word youth friendly services is a place that gives enjoyment to youth or organizes activities.” (Participant 4)

Also for “antenatal care,” the adolescents understood it to mean “rely on somebody to take care of baby or pregnancy,” so they thought it was to take the baby to bed for a rest or give the baby to someone else, as in adoption. Therefore, the questions were reformulated to “living healthy during a pregnancy and receiving (professional) help.” Finally, a consensus on adequate semantic equivalence was reached during the final expert meeting.

## Operational Equivalence

### Layout and Answering Scale

Both experts and adolescents were satisfied with the layout of the questionnaire; only minor adaptations were made. The answer option *I don't know/not sure* was removed, as both experts and adolescents agreed that it was not a practical addition to a scale from *easy to difficult* and that the questions were not asking about knowledge of individuals, but rather about their skill (to access, understand, appraise, and apply).

### Administration Mode

Both experts and adolescents preferred the interviewer-administered mode. Experts argued that a self-administered questionnaire might be difficult to understand for adolescents, especially for those who had left school. The interviewers must be trained to give an accurate and standardized explanation to adolescents during the interview, emphasizing not only their privacy, but also the wordings or terms that adolescents feel comfortable to answer. Adolescents also preferred the interviewer-administered mode, as it provides the opportunity to ask for an explanation. As one adolescent said: “I prefer an interview instead of reading the questions myself [. . .]. I did not feel sure about 5% of the questions. If someone interviews me, I am almost 100% sure to answer the questions correctly, . . . and I do not feel sleepy or that it feels like an examination.” (Participant 5)

### Measurement Equivalence

***Calculation of the index.*** The SRHL index was set from 0 to 50 based on the report of HLS-EU-Q47 (Pelikan et al., 2012). The calculation formula was (mean −1) × 50 divided by 3; the mean is the average score of all items; 1 is the smallest value of means, 3 is the interval of means, and 50 is the highest value set for the index. Both original and adapted versions of the questionnaire had similar index calculation formulas, whereas the adapted version better fit the formula as it lacked the *Don't know* option, which is impractical for scoring.

***Internal consistency.*** This was based on the two pilot testing surveys of the new questionnaire. There was high internal consistency. A strong reliability value of internal consistency between the items of the questionnaire was detected, with Cronbach's alpha ranges between 0.8 and 0.9 (**Table 3**).

**Figure 2.**
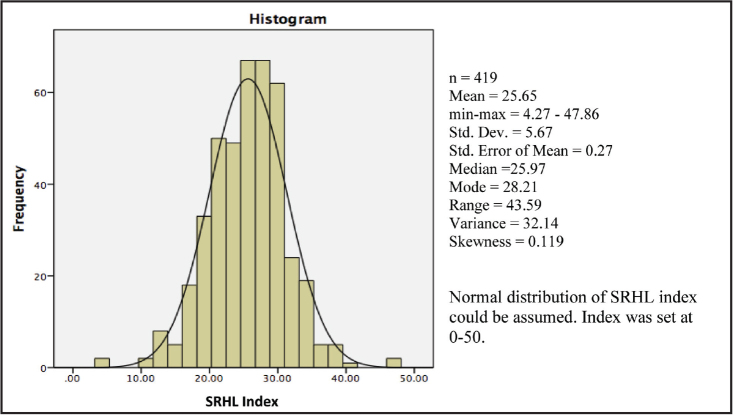
The distribution of the sexual and reproductive health literacy index. Dev = development; minmax = minimum-maximum; Std = standard; SRHL = sexual and reproductive health literacy.

***Floor and ceiling effect.*** There were no missing items, as the interviewer could help explain in case adolescents did not directly understand any items, Normal distribution of data were found in both tests, without floor and ceiling effects. The distribution of SRHL item responses was clearly achieved in both pilots (see S3 and S4: https://bit.ly/SRHL2022). A normal distribution of the SRHL index was assumed by considering that the skewness of data distribution was close to zero, and the values of mean and median of the index were relatively equal, without floor and ceiling effects. Participants had a minimum index of 4.2 of 50 and the maximum was 47.8 of 50 (**Figure 2**).

**Figure 3.**
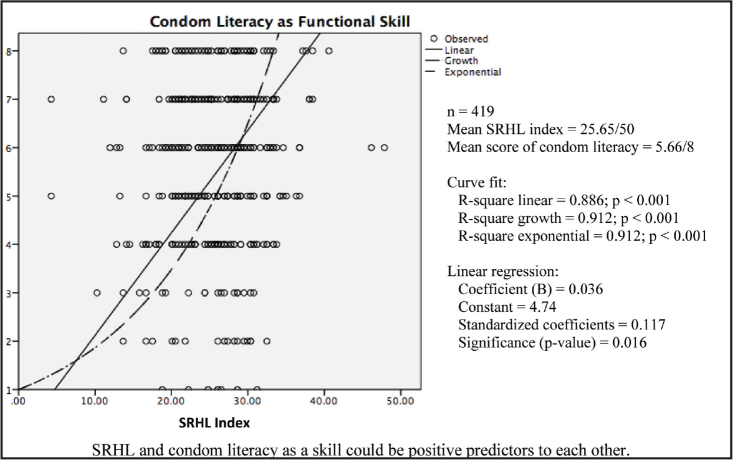
The interaction between sexual and reproductive health literacy index and condom literacy as a functional skill. SRHL = sexual and reproductive health literacy.

***Construct validity.*** In the final pilot, 6 of 7 statistical hypotheses were shown to be valid, except for the TP-related knowledge, which was not well correlated with the SRHL index (**Table 3**). **Table 4** shows a strong positive correlation between the index of different dimensions of SRHL (*p* < .01). A positive correlation was also found between the SRHL index and the score of the condom literacy skill, which means that the functional literacy skill on condom could be a positive predictor of the SRHL (**Figure 3**).

**Table 4:**
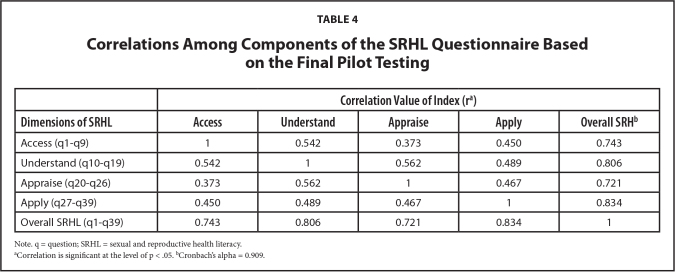
Correlations Among Components of the SRHL Questionnaire Based on the Final Pilot Testing

	**Correlation Value of Index (r^a^)**				
**Dimensions of SRHL**	**Access**	**Understand**	**Appraise**	**Apply**	**Overall SRH^b^**
Access (q1–q9)	1	0.542	0.373	0.450	0.743
Understand (q10–q19)	0.542	1	0.562	0.489	0.806
Appraise (q20–q26)	0.373	0.562	1	0.467	0.721
Apply (q27–q39)	0.450	0.489	0.467	1	0.834
Overall SRHL (q1–q39)	0.743	0.806	0.721	0.834	1

Note. q = question; SRHL = sexual and reproductive health literacy.

a Correlation is significant at the level of p < .05.

b Cronbach's alpha = 0.909.

## Discussion

This study aimed at the development and validation of a context appropriate SRHL questionnaire to assess adolescents' self-perceived capacity to manage their sexual and reproductive health. We used a rigorous process of quality testing, inspired by a range of tool validation reports (cited in the Methods section). We included both experts and targeted users in a mixed-method validation. The two-pilot testing confirmed that the adaptations increased the questionnaire validity. Four major improvements were made: (1) the focus was narrowed down to TP, contraceptives and abortion, leading to a new name, the SRHL questionnaire (SRHL-Q); (2) the administration mode was adjusted to interviewer-administered instead of self-administered; (3) the revised SRHL-Q consists of 39 items using a 4-point Likert scale (*very difficult*, *difficult*, *easy*, *very easy*) to assess the HL level of Lao adolescents on TP, contraceptives and abortion; and (4) good internal consistency was detected in the revised version, indicating that the items correlated highly with each other and measured the same thing. The results demonstrated an adequate cultural equivalence of the revised tool.

The domains of SRH include “physical, mental and social aspects of well-being related to the reproductive system” (UNFPA, 2018a). To reach an adequate SRH status, people need “to have a positive and respectful approach to sexuality and sexual relationships, as well as the possibility of having pleasurable and safe sexual experiences, free of coercion, discrimination and violence” (WHO, 2017a). They need “to have a responsible, satisfying and safe sex life and to have the capability to reproduce and the freedom to decide if, when and how often to do so” (WHO, 2017b). From those definitions, measuring the literacy regarding SRH in individuals would be difficult and might need long questions or a more complex tool.

In the current study, instead of expanding the questionnaire to cover all SRH domains, we chose to narrow the scope of the questionnaire to TP, contraceptives, and abortion to reflect the most fundamental SRH needs of adolescents in Laos. This was also justified because TP remains a major social and health concern in Laos; maternal death among girls age 14 to 18 years rose from 7% from 2011 to 2013 to 10% in 2014 to 2016 (UNFPA, 2018b). A focused questionnaire helps to measure the capacity or skill of adolescents to deal with SRH issues, whereas several health and education programs are trying to promote SRH knowledge to reduce TP.

Based on inputs from both experts and adolescents, it was highly recommended to administer the questionnaire face-to-face instead of the often-used self-administered approach. Response bias is more likely to occur in self-administered questionnaires, compared with interviewer-administered questionnaires, partly due to low response rate among people with low literacy/educational level (Czaja & Blair, 2005). The interviewer-administered mode allows for probing questions, which increases the validity of the answers. Personal contact increases the participation and the response rate, thus avoiding missing information (Gray, 2014). The interviewer-administered mode might, however, have challenges, such as costing more time and money, plus the possibility of interviewer bias (Gray, 2014). There is reduced anonymity for respondents. Adolescents might feel uncomfortable about sharing sensitive information (for example on abortion) with the interviewer face to face. Interviewees might also be tempted to respond in a more socially desirable way. Yet, we found at least one study showing that an interviewer-administered mode was not associated with more socially desirable answers compared with a self-administered mode (Hancock & Flowers, 2001). That means that in some cases, interviewer-administration might be practical, when the target users prefer it. To reduce the chances of interviewer bias and socially desirable answers, it is recommended to train the interviewers about how to establish rapport and to probe in case of unclear answers. Interviewers need to use everyday language with adolescents. Before using this tool in a different context, a small pilot is recommended before employing the measurement on a larger scale.

In the answer scale, the option *I don't know* was deemed unnecessary. In a previous study, this choice was suspected of causing a low value of item responses and suggesting a low SRHL level; respondents who had low knowledge might select only *I don't know*, which might be inaccurate (Vongxay et al., 2019). Or if their answer did not fit a question's requirement, they can leave the question blank, which leads to low frequency of valid responses or a high frequency of missing responses (Beatty et al., 1998; Vongxay et al., 2019). Answering *I don't know* and *very difficult* were suggested to be very similar (for example, either might apply to an adolescent girl unfamiliar with abortion information and finding it very difficult to answer). The *I don't know* option was therefore considered unnecessary in the SRHL-Q, similarly to the case of the HLS-EU-Q47, which was validated in six Asian countries (Duong et al., 2017; Sørensen, et al., 2015).

This study indicates that the SRHL-Q is valid and reliable to use with adolescents, based on current research of its equivalences. However, there are still doubts as to whether HL can in fact be measured using a subjective measurement approach that rates self-perceived ease or difficulty in managing health-related tasks. Similar to the HLS-EU-Q47, the SRHL-Q measures the perceived ease or difficulty of adolescents in performing health-related tasks (Pelikan et al., 2012). A more profound investigation might need more mixed methods, such as an objective and a subjective measurement (Altin et al., 2014).

Based on Pelikan et al. (2012), the HL index of 0 to 50 was classified into four levels: index of index of 0 to <25 as inadequate, index of 25 to <33 as problematic, index of 33 to <42 as sufficient, and index of 42 to 50 as excellent. In this study, with the adapted questionnaire, the mean index of SRHL of adolescents was 25.6, which is at the problematic level. It was, however, higher than the index in a previous study in Laos, using the SRHL-Q in the original version, which was only 19.2, which is the level of inadequate (Vongxay et al., 2019). The index based on the adapted version seems more realistic for adolescents, especially since SRH programs have been introduced both in schools and in the community for some years. The SRHL-Q works better without the option *don't know/not sure*.

The extended analysis of statistical hypotheses testing has emphasized another strength of the questionnaire's construction and psychometrics, because 6 of 7 hypotheses (>75% of overall result) were confirmed as expected (Terwee et al., 2007). The questionnaire could be applicable in further adolescent reproductive health surveys, regardless of gender, level of knowledge and sexual experience. However, the association between SRHL and TP-related knowledge in the re-testing was found to be weaker than in a previous study (Vongxay et al., 2019). That result might be due to using a different set of knowledge questions (Inthapanyo, 2019), or due to different samples, or both. Future studies might need to reconsider the construction of the questionnaire for assessing TP-related knowledge and to retest how it would interact with the SRHL.

Currently, there is no clear consensus on what is a representative measure for HL (Altin et al., 2014; Haun et al., 2014; Pleasant & McKinney, 2011). Should all domains of HL be measured in one single questionnaire, or should different domains be measured separately? Based on our findings, we agree with Pleasant & McKinney (2011) that HL measurements should include a broad definition and conceptualization, especially as all domains of HL together affect behavior (Pleasant & McKinney, 2011; Sørensen et al., 2012). For example, adolescents may have knowledge about the advantages and disadvantages of contraceptives, but if they do not have the communication and/or application skills, they will not use that knowledge.

Peters et al. (2014) provide insights into psychometric properties, including internal consistency, construct validity, agreement, reliability, floor and ceiling effects, and interpretability for the measurement of equivalence. The current study emphasizes specifically the consistent calculation of measurement problems, the internal consistencies of measurements between two different groups of samples, ability to assume normal distribution of the data, without floor and ceiling effects, and the construct validity, using multiple hypotheses. This could be another way to look at whether a validation of a tool needs to investigate different groups of samples while measuring the same thing.

This research highlights the importance of validating a questionnaire and aligning those measures that fit the context. The original questionnaire was adapted quite extensively based on the feedback from experts and adolescents collected during this study. By adaptations, such as contextualizing the questions and using the everyday language of Lao adolescents, the SRHL-Q became much clearer, which is important to ensure accurate data collection.

## Study Limitations

The study was done only in the province of Vientiane, Lao PDR, which could be a limitation in representation, as it is only 1 of 18 provinces in the country. However, we did make efforts to ensure representation for adolescents both in-school and out-of-school, and from both urban and rural communities. Furthermore, information related to abortion is limited in Laos, which might have contributed to the low level of SRHL. However, it is important to keep the abortion domain in the questionnaire, as the experts suggested that it also reflects adolescents' self-perceived ease or difficulty communicating about sensitive topics. Another limitation is that the items are not comprehensive; they do not assess all skills related to TP (e.g., conversations with partners about using contraceptives). Items were designed for ease of answering by adolescents, based on perception of their skill, regardless of whether they have ever had sex. We were also limited in the way we could assess functional literacy (e.g., regarding use of condoms). As we were not allowed to ask the participants to demonstrate the use of a condom, we used a proxy approach, asking them to handle the package and checking whether they were able to answer questions about it. We feel that this at least reflects their familiarity with and knowledge about how to use a condom. This technique was also consistent with a previous study that asked questions about whether the text on the package is effective to finding out if people can understand what they read (Weiss et al., 2005).

## Conclusion

The SRHL-Q was carefully developed through conceptual, item, semantic, operational and measurement equivalence. All equivalences indicated good cross-cultural validity. Yet, HL is a complex concept and to date, there is no consensus on a representative measurement instrument. Context-specific adaptation with the support of local experts and inputs from the target population should be required for this type of measurement in further research in other contexts. This tool appeared to be effective in determining the level of SRHL in adolescents, focusing on TP and related issues.

## Implications and Contribution

This study contributes to the development of a valid questionnaire to measure HL among adolescents regarding TP, contraceptives, and abortion. It highlights the importance of cross-cultural validity. Beyond the knowledge, the measurement can serve for investigation of adolescents' self-assessment of their capacity to access, understand, appraise, and apply information to lead to safer decision-making for their own sexual and reproductive health.
